# Combined Use of Delamanid and Bedaquiline to Treat Multidrug-Resistant and Extensively Drug-Resistant Tuberculosis: A Systematic Review

**DOI:** 10.3390/ijms18020341

**Published:** 2017-02-07

**Authors:** Giovanni Battista Migliori, Emanuele Pontali, Giovanni Sotgiu, Rosella Centis, Lia D’Ambrosio, Simon Tiberi, Marina Tadolini, Susanna Esposito

**Affiliations:** 1 World Health Organization Collaborating Centre for Tuberculosis and Lung Diseases, Maugeri Institute, IRCCS Tradate 21049, Italy; giovannibattista.migliori@icsmaugeri.it (G.B.M.); rosella.centis@icsmaugeri.it (R.C.); liadambrosio59@gmail.com (L.D.); 2 Department of Infectious Diseases, Galliera Hospital, Genoa 16128, Italy; pontals@yahoo.com; 3 Clinical Epidemiology and Medical Statistics Unit, Department of Biomedical Sciences, University of Sassari, Sassari 07100, Italy; gsotgiu@uniss.it; 4 Public Health Consulting Group, Lugano CH-6904, Switzerland; 5 Division of Infection, Royal London Hospital, Barts Health NHS Trust, 80 Newark Street, London E1 2ES, UK; simon.tiberi@bartshealth.nhs.uk; 6 Unit of Infectious Diseases, Department of Medical and Surgical Sciences, Alma Mater Studiorum University of Bologna, Bologna 40138, Italy; mtadolini@hotmail.com; 7 Pediatric Clinic, Department of Surgical and Biomedical Sciences, Università degli Studi di Perugia, Perugia 06129, Italy; 8 Pediatric Highly Intensity Care Unit, Fondazione IRCCS Ca’ Granda Ospedale Maggiore Policlinico, Milan 20122, Italy

**Keywords:** MDR-TB, XDR-TB, delamanid, bedaquiline, effectiveness, safety, tolerability

## Abstract

The new drugs delamanid and bedaquiline are increasingly being used to treat multidrug-resistant (MDR-) and extensively drug-resistant tuberculosis (XDR-TB). The World Health Organization, based on lack of evidence, recommends their use under specific conditions and not in combination. No systematic review has yet evaluated the efficacy, safety, and tolerability of delamanid and bedaquiline used in combination. A search of peer-reviewed, scientific evidence was carried out, aimed at evaluating the efficacy/effectiveness, safety, and tolerability of delamanid and bedaquiline-containing regimens in individuals with pulmonary/extrapulmonary disease, which were bacteriologically confirmed as M/XDR-TB. We used PubMed to identify any relevant manuscripts in English up to the 23 December 2016, excluding editorials and reviews. Three out of 75 manuscripts retrieved satisfied the inclusion criteria, whilst 72 were excluded for dealing with only one drug (three studies), being recommendations (one study) or identifying need for their use (one study), focusing on drug resistance aspects (six studies) or being generic reviews/other studies (61 papers). The studies retrieved reported two XDR-TB cases observed for six months and achieving consistent sputum smear and culture conversion. Case 2 experienced a short break of bedaquiline, which was re-started after introducing verapamil. After a transient and symptom-free increase of the QT interval from week 5 to 17, it then decreased below the 500 ms threshold.

## 1. Introduction

According to the World Health Organization (WHO), tuberculosis (TB) was responsible for over 10.4 million cases of disease in 2015, 480,000 of whom were affected by multidrug-resistant (MDR)-TB (10% meeting the criteria for extensively drug-resistant (XDR)-TB) and 100,000 by rifampicin-resistant TB, all contributing to an estimated 190,000 deaths [1,2,3,4].

The challenges of treating these cases are well known by clinicians operating in MDR-/XDR-TB reference centres, due to the long duration of treatment, the cost of expensive regimens, poor drug tolerability associated with frequent adverse events, and a high treatment failure rate [3,4,5,6,7,8,9,10]. As a result, as of today not all diagnosed MDR-TB cases have had access to quality treatment [1].

In 2016 the approach to design MDR-TB regimens has changed from the original stepwise approach based on five groups of drugs in priority order [9,11] to a new approach based on the new drugs’ classification [7,12,13].

The new classification includes delamanid and bedaquiline in group D2 [7], their use is recommended under specific conditions in adults (e.g., for six months, at the recommended doses in addition to an optimised background regimen, in the presence of pharmacovigilance and informed consent, under adequate clinical and QT interval monitoring) [14,15,16]. Delamanid use was recently approved for children above six years of age [16,17].

Delamanid and bedaquiline have been proven to be effective in increasing sputum smear and culture conversion, and in improving success rates at the end of treatment, although concerns exist regarding the possible cardiotoxicity resulting from drug interactions with other drugs known to increase the QT interval (the measure of time between the start of the Q wave and the end of the T wave in the heart’s electrical cycle), such as fluoroquinolones and clofazimine [18,19,20,21,22,23,24,25,26].

Bedaquiline (previously known also as TMC207 or R207910) belongs to the diarylquinoline group, and it was developed by Janssen Pharmaceuticals (Titusville, NJ, USA) [27]. This is the first anti-TB drug discovered and developed in its class. Structurally, it presents a quinolinic central heterocyclic nucleus with alcohol and amine side chains that are the key effectors for its antimycobacterial activity. So far, bedaquiline acts by inhibiting the mycobacterial ATP synthase, thus being the first and only anti-TB drug targeting the energy metabolism of mycobacteria [18]). Bedaquiline is characterised by long half-life well exceeding 24 h. Clinical studies have evidenced good early bactericidal activity, safety, tolerability, and pharmacokinetic profile of bedaquiline; this has led to early accelerated or conditional approval in the USA (2012) and Europe (2014) for use in MDR-TB [18].

Delamanid (previously known also as OPC-67683) is a nitro-dihydro-imidazooxazole derivative belonging to the class of nitroimidazoles. It was developed and produced by Otsuka Pharmaceutical Development and Commercialization (Osaka, Tokyo, Japan) [19]. The mycobacterial wall is well known to be wax-rich; one of the key components of mycobacterial wax is mycolic acid. The molecular mechanism of action of delamanid and its class is based on its capacity to inhibit the biosynthesis of mycolic acid. During its development delamanid showed a brilliant in-vitro and in-vivo activity, being efficacious against both extra- and intracellular mycobacteria. Clinical studies showed good tolerability in the absence of frequent severe adverse events with a non-clinically significant effect on the QTc interval [19]. Although there are other promising drugs in this class, such as PA-824, delamanid is the first drug in its class to reach approval and clinical use.

The difficult task for any clinician dealing with MDR-TB is to ensure the minimum number of drugs necessary to design an effective regimen [2,7,10,13,28].

In this regard, as of today, some repurposed drugs can be used including, among others, linezolid [29,30,31,32,33,34,35] and the carbapenems [36,37,38,39,40,41].

However, when patients have drug resistance profiles “beyond XDR-TB” [3,4] or intolerance to second-line drugs so that four active drugs are not available, clinicians may be forced to combine delamanid and bedaquiline [41].

Considering the forecasted harms and potential benefits of this drug combination, a review of the available scientific evidence on this topic is of crucial importance.

The aim of the present manuscript is to perform a systematic review on the combined use of delamanid and bedaquiline.

## 2. Methods

We conducted a search of peer-reviewed, scientific evidence to evaluate the efficacy/effectiveness, safety, and tolerability of regimens containing delamanid and bedaquiline in individuals with pulmonary/extrapulmonary TB which was culture- and drug susceptibility testing (DST)-confirmed as M/XDR-TB.

The database PubMed was used to identify any manuscript, without any time constraints up until 23 December 2016. Conference abstracts were excluded on the basis of their limited word count, the information provided was considered insufficient to assess the objectives described above. Letters or case series/reports including detailed clinical information were considered. Only papers written in English were analysed.

The keywords TB, delamanid, and bedaquiline were used to be as inclusive as possible.

The following search exclusion criteria were used:

1 Experimental studies on animals with TB;
2 Reviews and editorials on delamanid and bedaquiline;
3 M/XDR-TB diagnosis of treated patients not confirmed with conventional bacteriological criteria.

Two authors independently performed the search and evaluated the titles and abstracts of the records according to the selection criteria. Potentially interesting articles were critically assessed; when they fulfilled the enrolment criteria, the planned information (e.g., sputum smear and culture conversion, treatment outcomes, adverse events and their grading, demographics, epidemiological data, drug resistance patterns of the collected *Mycobacterium tuberculosis* isolates, drug regimen prescribed and its duration) was retrieved and collected using a pre-defined electronic template. The study was conducted following the guidelines of the 2009 PRISMA (Preferred Reporting Items for Systematic Reviews and Meta-Analysis) statement [42].

## 3. Results

**Figure 1:**
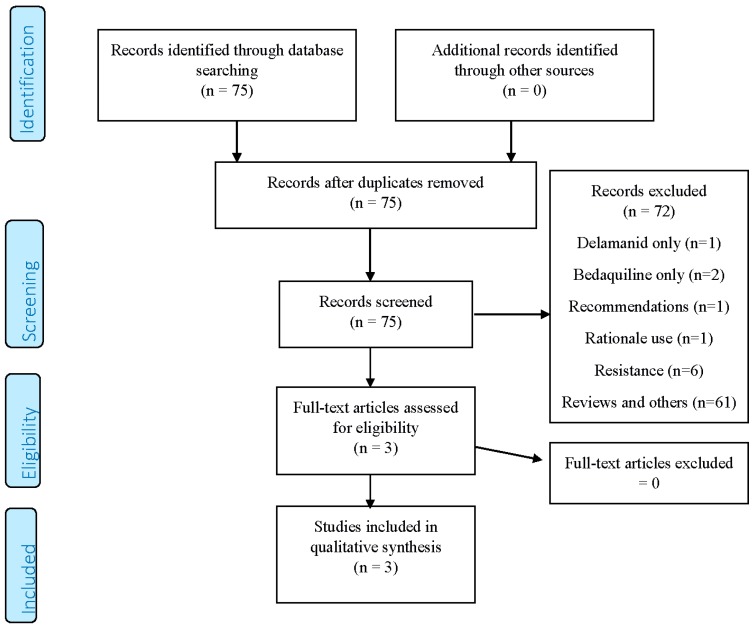
PRISMA (Preferred Reporting Items for Systematic Reviews and Meta-Analysis) 2009 Flow Diagram.

A total of 75 records were obtained from the search (PRISMA flowchart, Figure 1); 72 were excluded for the following reasons: dealing with only one of the two drugs (three studies), being recommendations on the individual or combined use (one document), identifying need for their use (one study), focusing on drug resistance aspects (six studies) or being generic reviews/other studies (61 papers).

Three studies [43,44,45] satisfied our criteria for further analysis, all of them being research letters published in 2016, and reporting a total of two cases. One study [45] updated another one [44], both being from the same authors.

The two cases were both XDR-TB and extremely complicated, one born in Africa and one in Asia, and exposed to the combination of delamanid and bedaquiline in 2015 and 2016, respectively.

For both of them, a study follow-up of six months is reported, with the achievement of sputum smear and culture conversion over the study period.

Only one case experienced the QT interval prolongation, followed by temporary discontinuation of bedaquiline, and re-administration after the prescription of verapamil [43].

Although the QT interval transiently increased from week 5 to week 17, it then decreased below 500 ms.

Details on the studies are reported in Table 1, Table 2 and Table 3.

## 4. Discussion

The aim of the present study was to systematically review the available scientific evidence on the combined use of delamanid and bedaquiline in M/XDR-TB patients.

Only three papers included information of cases (two) treated with a combination of the two new drugs.

The information is scarce due also to the preliminary format used (letter) which does not allow for the inclusion of detail. The main conclusions which can be drawn from this analysis are the following:

1. In extremely challenging M/XDR-TB cases, when the number of drugs are not enough to reach the recommended number of at least four to design an effective regimen, some clinicians have considered using delamanid and bedaquiline in combination.
2. The combination was effective in the two cases observed as it achieved smear and culture conversion, but their number is insufficient to draw any firm conclusions.
3. The combination of delamanid and bedaquiline, and, eventually, of other QT interval-prolonging drugs (e.g., fluoroquinolones, clofazimine) is prone to adverse events and potentially harmful QT prolongation [41,46]. The recommendation to obtain and assess a baseline electrocardiogram (ECG) prior to starting the combination treatment and regularly repeat ECGs during treatment with such drugs to monitor the QT interval is not just ‘formal’, but clinically relevant. ECG should be performed at baseline and, then, at regular intervals (e.g., weekly on the first instance and in reduced frequency should QTc prolongation not manifest).
4. Electrolytes (potassium and magnesium) as well as albumin should be monitored as electrolyte disturbance and/or hypoalbuminemia (delamanid) may precede QTc prolongation.
5. Given the arguments above, only specialised centres should manage patients with delamanid-bedaquiline combined treatment, according to the criteria proposed by Matteelli A. et al. [41].
6. As the global experience with combined delamanid-bedaquiline treatment is still very limited, new clinical trials are needed to assess the real efficacy, safety, and tolerability of these drugs in TB cases with complicated drug-resistance patterns.
7. Interestingly, both patients made a good clinical improvement on treatment. Surgery was performed on the Congolese patient (who likely benefited from it). However, the Tibetan patient could not afford an operation having bilateral lesions at the chest radiography. The combination regimen may be surgery-sparing; it could be utilised in patients with relative or absolute contraindications for surgery. [43,44,45].
8. No published evidence is available in children yet, although clinically-based recommendations have been recently published in this sense [17] and a recent study by Medecins Sans Frontieres (MSF) demonstrates the real need to increase the availability of new drugs [47]. Interestingly, a preliminary report from the MSF projects presented in Liverpool on 24 cases [48] seems to be encouraging.

More experience with these two agents is, therefore, needed. Two clinical trials NCT02583048 (recruiting) and NCT02754765 (planned) [27] may shed more light on this combination and any possible major interactions. Preliminary data from the first aforementioned trial may be available by April 2017. Moreover, NCT02583048 is recruiting from only one of three sites and with an estimated enrollment of 84 patients. It is unlikely that we will have trial-based safety data anytime soon.

Another nitroimidazole, pretomanid, appears to work in synergy with bedaquiline [49], therefore excluding major class effects.

In conclusion, more evidence is needed on the combined use of delamanid and bedaquiline.

## Figures and Tables

**Table 1:** Epidemiological characteristics of the selected studies.

First Author	Publication Year	Country	Study Design	Clinical Setting	Study Duration
Lachâtre M. [43]	2016	France	Case 1 Report	Reference Hospital, Paris, France	March–September 2015
Tadolini M. [44]	2016	India	Case 2 Report	Reference Hospital, Dharamshala, India	February–March 2016
Tadolini M. [45]	2016	India	Case 2 Report update	Reference Hospital, Dharamshala, India	February–July 2016

**Table 2:** Clinical features of the selected studies.

First Author	Number of Individuals Exposed to Delamanid + Bedaquiline/HIV Status	Age (Years)/Sex/Country of Birth	XDR-TB/Previous Relapses	Body Weight at the Start of Treatment	Drug Resistance Profile	Regimen Administered (Doses if Available)
Lachâtre M. [43]	1, HIV ND	20/male/DR Congo	Yes/ND	ND	ND	delamanid, bedaquiline, ethambutol, para-aminosalicylic acid, linezolid, imipenem, amoxicillin/clavulanate
Tadolini M. [44]	1, HIV negative	39/female/Tibet	Yes/2	65 kg	Resistant to 12 drugs: isoniazid, rifampicin, kanamycin, amikacin, capreomycin, moxifloxacin, ofloxacin, ethionamide, Para-amino-salicylic acid, linezolid, high dose isoniazid, moxifloxacin, Susceptible to: clofazimine	delamanid (200 mg), bedaquiline (400 mg), clofazimine (200 mg), terizidone (1 g), meropenem 1g TID, amoxicillin/clavulanate 1 g/200 mg TID i.v.)
Tadolini M. [45]	same as above	same as above	same as above	same as above	same as above	

TID: Thrice a day, i.v.: intravenously; ND: Not Declared; XDR-TB: extensively drug-resistant tuberculosis.

**Table 3:** Effectiveness, safety, and tolerability profiles of delamanid and bedaquiline combination in the selected studies.

First Author	Sputum Smear Conversion	Sputum Culture Conversion	Treatment Outcome	QT Interval Prolongation	Interruption of Bedaquiline or Delamanid due to Adverse Events
Lachâtre M. [43]	Yes	Yes	After six months favourable clinical, microbiological, and radiological responses	No	ND
Tadolini M. [44]	Yes	Yes	After two months favourable clinical, microbiological, and radiological responses	Yes	Bedaquiline stopped on 7th March 2016 restarted on 12 March 2016
Tadolini M. [45]	Yes (consistent negative sputum smear)	Yes (consistent negative culture)	After six months favourable clinical, microbiological, and radiological responses. Body weight increased 4 kg.	Yes, W5: 508 ms; W7: 500 ms; W8: 508 ms; W12: 512 ms; W13: 510 ms; W15: 507; W16: 520 ms; W17: 501 ms	Not after 12 March 2016 and addition of verapamil

ND: Not Declared; W: week; ms: milliseconds.
